# m-RESIST, a Mobile Therapeutic Intervention for Treatment-Resistant Schizophrenia: Feasibility, Acceptability, and Usability Study

**DOI:** 10.2196/46179

**Published:** 2023-06-30

**Authors:** Eva Grasa, Jussi Seppälä, Anna Alonso-Solis, Marianne Haapea, Matti Isohanni, Jouko Miettunen, Johanna Caro Mendivelso, Cari Almazan, Katya Rubinstein, Asaf Caspi, Zsolt Unoka, Kinga Farkas, Judith Usall, Susana Ochoa, Shenja van der Graaf, Charlotte Jewell, Anna Triantafillou, Matthias Stevens, Elisenda Reixach, Jesus Berdun, m-RESIST group, Iluminada Corripio

**Affiliations:** 1 Mental Health Institut d’Investigació Biomèdica Sant Pau (IIB SANT PAU) Barcelona Spain; 2 Centro de Investigación Biomédica en Red de Salud Mental Instituto de Salud Carlos III Madrid Spain; 3 Social Insurance Institution of Finland Kuopio Finland; 4 Research Unit of Population Health University of Oulu Oulu Finland; 5 Mental Health Division Fundació Althaia Xarxa Assistencial Universitaria de Manresa Manresa Spain; 6 Medical Research Center Oulu Oulu University Hospital and University of Oulu Oulu Finland; 7 Department of Psychiatry Oulu University Hospital Oulu Finland; 8 Agency for Health Quality and Assessment of Catalonia (AQuAS) Barcelona Spain; 9 The Gertner Institute of Epidemiology and Health Policy Research Sheba Medical Center Tel-Aviv University Tel-Aviv Israel; 10 Department of Psychiatry and Psychotherapy Semmelweis University Budapest Hungary; 11 Parc Sanitari Sant Joan de Déu Sant Boi de Llobregat Spain; 12 Etiopatogènia i tractament dels trastorns mentals greus (MERITT) Institut de Recerca Sant Joan de Déu Esplugues de Llobregat Spain; 13 Department of Communication Science University of Twente Twente Netherlands; 14 Lentic-CRIS University of Liège Liège Belgium; 15 Athens Technology Center Athens Greece; 16 EDiT Department imec Ghent/Antwerp Belgium; 17 Solutions Department imec Leuven Belgium; 18 TicSalut Health Department Generalitat de Catalunya Barcelona Spain; 19 Digital Health Unit Hospital de la Santa Creu i Sant Pau Institut d’Investigació Biomèdica Sant Pau (IIB SANT PAU) Barcelona Spain; 20 see Authors' Contributions; 21 Mental Health and Psychiatry Department Vic Hospital Consortium Vic Spain

**Keywords:** schizophrenia, treatment-resistant, digital mental health, mHealth, mobile health, mental health, mental illness, mental disorder, psychosis, symptom management, adherence, acceptability, usability, feasibility, digital intervention, mobile intervention, mobile phone

## Abstract

**Background:**

In the European Union, around 5 million people are affected by psychotic disorders, and approximately 30%-50% of people with schizophrenia have treatment-resistant schizophrenia (TRS). Mobile health (mHealth) interventions may be effective in preventing relapses, increasing treatment adherence, and managing some of the symptoms of schizophrenia. People with schizophrenia seem willing and able to use smartphones to monitor their symptoms and engage in therapeutic interventions. mHealth studies have been performed with other clinical populations but not in populations with TRS.

**Objective:**

The purpose of this study was to present the 3-month prospective results of the m-RESIST intervention. This study aims to assess the feasibility, acceptability, and usability of the m-RESIST intervention and the satisfaction among patients with TRS after using this intervention.

**Methods:**

A prospective multicenter feasibility study without a control group was undertaken with patients with TRS. This study was performed at 3 sites: Sant Pau Hospital (Barcelona, Spain), Semmelweis University (Budapest, Hungary), and Sheba Medical Center and Gertner Institute of Epidemiology and Health Policy Research (Ramat-Gan, Israel). The m-RESIST intervention consisted of a smartwatch, a mobile app, a web-based platform, and a tailored therapeutic program. The m-RESIST intervention was delivered to patients with TRS and assisted by mental health care providers (psychiatrists and psychologists). Feasibility, usability, acceptability, and user satisfaction were measured.

**Results:**

This study was performed with 39 patients with TRS. The dropout rate was 18% (7/39), the main reasons being as follows: loss to follow-up, clinical worsening, physical discomfort of the smartwatch, and social stigma. Patients’ acceptance of m-RESIST ranged from moderate to high. The m-RESIST intervention could provide better control of the illness and appropriate care, together with offering user-friendly and easy-to-use technology. In terms of user experience, patients indicated that m-RESIST enabled easier and quicker communication with clinicians and made them feel more protected and safer. Patients’ satisfaction was generally good: 78% (25/32) considered the quality of service as good or excellent, 84% (27/32) reported that they would use it again, and 94% (30/32) reported that they were mostly satisfied.

**Conclusions:**

The m-RESIST project has provided the basis for a new modular program based on novel technology: the m-RESIST intervention. This program was well-accepted by patients in terms of acceptability, usability, and satisfaction. Our results offer an encouraging starting point regarding mHealth technologies for patients with TRS.

**Trial Registration:**

ClinicalTrials.gov NCT03064776; https://clinicaltrials.gov/ct2/show/record/NCT03064776

**International Registered Report Identifier (IRRID):**

RR2-10.1136/bmjopen-2017-021346

## Introduction

Schizophrenia is a severe mental disorder that affects approximately 1% of the population worldwide [1]. The results of a meta-analysis showed that only 13.5% of the patients achieve recovery, which includes clinical remission and good social functioning outcome [2]. Despite the proven efficacy of antipsychotic drugs, 30%-50% of patients with schizophrenia obtain a scarce benefit from conventional treatments [3,4]. This inadequate response to pharmacological treatment is known as treatment-resistant schizophrenia (TRS). TRS implicates an important burden at 3 levels, as follows: (1) clinical: negative attitude to medication, drug abuse, and nutritional/physical health problems; (2) economic: hospitalizations and polytherapy; and (3) humanistic: depression and social isolation [5]. As a result, dealing with TRS involves a high emotional burden for patients and their caregivers, affecting their quality of life. The shortcomings of the current health care and social support systems cannot provide adequate and effective solutions to these patients.

During the last decade, the incorporation of information and communication technology (ICT) into health care services (eHealth) has led to developing interventions aimed to improve patients’ quality of life. In the case of TRS, ICT tools could offer a novel opportunity to overcome important barriers by (1) enabling tailored therapeutic processes, (2) improving accessibility and continuous integrated care, and (3) promoting patients’ empowerment and participation of informal caregivers in therapeutic processes. Results from earlier studies on psychosis [6-8] support the idea of including ICT tools in the treatment of patients with TRS. Moreover, smartphone ownership among people with schizophrenia is relatively high and increasing [9-11]. Patients seemed willing and able to use smartphones to monitor their symptoms, engage in therapeutic interventions, and increase physical exercise [12]. Feasibility studies previous to m-RESIST have shown that interventions based on mobile health (mHealth) could promote the empowerment of patients with schizophrenia [9,13]. Further, some mHealth interventions may be effective for increasing treatment adherence, decreasing relapses, and improving symptoms [6,14,15].

To the best of our knowledge, none of the previously published studies on mHealth has focused on TRS [16]. We aimed to present the results of a 3-month prospective multicenter feasibility study performed as part of the m-RESIST project, wherein a new hybrid program (digital interventions along with face-to-face strategies) using wearable computing solutions and offering high modular and flexible functioning was designed and developed.

This study aims to assess the feasibility, acceptability, usability, and satisfaction of patients with TRS after using the m-RESIST intervention. We hypothesized that the m-RESIST intervention would have acceptable rates of willingness to enroll (≥70%) and attrition (nonusage and dropout attrition <15% in both measures) and would be highly accepted by patients with TRS, showing high scores in acceptability, usability, and satisfaction reported by more than 80% of the sample.

## Methods

### Study Design

A prospective multicenter feasibility study without a control group was performed in patients with a diagnosis of TRS between March and November 2017. Participants were recruited from 3 clinical sites: Sant Pau Hospital (Barcelona, Spain), Semmelweis University (Budapest, Hungary), and Sheba Medical Center and Gertner Institute of Epidemiology and Health Policy Research (Ramat-Gan, Israel). Patients underwent a 3-month-long modular intervention, which was specifically designed for patients with TRS. The intervention was based on the recognition of early warning signs of psychosis in order to improve positive symptoms, treatment adherence, and healthy lifestyle (Multimedia Appendix 1). To deliver the m-RESIST intervention and to promote active participation in the therapeutic processes, 3 mHealth tools were included: a wearable device (smartwatch), a mobile app (m-RESIST app), and a web-based platform. The m-RESIST intervention was created to be provided in 5 languages: Spanish, Catalan, Hungarian, Hebrew, and English. Mental health care providers assisted patients with TRS in using the intervention. The trial was registered in Clinical Trials (NCT03064776), and the study protocol was published earlier [17].

### Ethics Approval

Ethics approval for this study was provided by the corresponding clinical ethics committees of the 3 participating clinical sites: Hospital Santa Creu i Sant Committee (IIBSP-RES-2016-51), ETT Tukeb, Semmelweis Ethical Committee (54920-4/2016/EKU), and Sheba Medical Center Ethical Committee (3472-16 SMC). Written informed consent was obtained from the participants before performing any study-related activities. The database generated by the study did not contain any identification of the participants but only a numerical code kept in a separate list; thus, the patients’ identities were protected. The information collected in this study was always treated as grouped data and never as individual or personal data, thereby maintaining anonymity and confidentiality.

### Study Participants

Patients with TRS were recruited for this study. Inclusion criteria were patients aged 18-45 years with a diagnosis of schizophrenia according to Diagnostic and Statistical Manual of Mental Disorders, fifth edition criteria; duration of disease less than 15 years to meet the criteria for TRS [18,19]; familiarity with ICT tools and physical capability to use them; and presence of a caregiver. Exclusion criteria were to meet criteria for remission according to the Remission of Schizophrenia Working Group [20]; presence of delusions mainly related to their therapists or with new technologies; presence of vision, hearing, or motor impairment, interfering with operating a smartphone; presence of a caregiver or informal carer who is not used to ICT tools or has physical incapability to use them; and presence of intellectual developmental disability. The caregivers were also recruited as part of the m-RESIST project. Their input about the functioning of the intervention was collected in focus groups. These data were not analyzed in this study.

A structured interview was administered to the patients, including sociodemographic data and ICT level measured with 5 grades (none, basic, average, good, excellent) for 3 questions: “How do you rate your current skills in using technological devices?,” “What devices do you own?,” and “For what purpose have you used them in the last 2 months?”

### Measures in This Study

#### Feasibility

Feasibility was assessed by analyzing patients’ willingness to participate and the rates of dropouts, nonuse, and compliance. For dropouts, the reasons and factors that could have prevented withdrawal were identified. A digital questionnaire (Qualtrics software) was used to present the following 3 questions: “Why have you decided to stop participating in the study? What was the main reason?,” “How could the m-RESIST intervention have been different to enhance your experience?,” and “Is there anything else that we haven’t discussed yet that you would like to mention?”

#### Acceptance and Usability

A mixed qualitative and quantitative methodological approach was used to analyze the outcomes. This study was performed following the principles of the 2 theoretical frameworks that best fit the evaluation of the m-RESIST intervention for acceptance and usability: Technology Acceptance Model (TAM [21]) and Living Labs or Ecosystem of Open Innovation [22].

The theoretical framework assumed to explore the acceptance of the m-RESIST intervention in patients with TRS was an extension of Davis’s model and based mainly on Chau and Hu’s model of telemedicine acceptance [23], which comprises 3 dimensions: technological (including perceived utility, perceived ease of use, habit), individual (attitude, barriers, intention to use), and organizational contexts (subjective norm, facilitators). All these variables were self-assessed by an adapted version to the field of mental health of the TAM scale [24], administered at the end of the study.

Usability was evaluated online in 2 different ways.

1. Measure of the degree of usability and experience after using the intervention: perceived ease of use, perceived utility, perceived quality of content, and attitude were assessed by the user experience questionnaire. The questionnaire was presented using the Qualtrics software. It consisted of 4 closed (4-level Likert scale) and 7 open questions. Data were collected in the middle and at the end of the study.
2. Measure of the continuous experience degree: the aim was to capture the continuous experience with the intervention. Through the app’s messaging system, the following question was sent to patients once a week and on different days and times: “What is it like to use the m-RESIST intervention?”

#### Satisfaction

User satisfaction was measured using the Client Satisfaction Questionnaire-8 item scale [25] to collect opinions about the service received. This scale contains 8 items with a range of scores from 1 to 4. This measure was self-assessed and collected at the end of the study.

#### Safety Measures

The presence of adverse events, defined as any clinical change or illness reported, was monitored using patient interviews throughout the study.

### Procedures, Stages, and Study Plan

This study was performed over 4 periods (for more details, see Alonso-Solís et al [17]): recruitment, preintervention, intervention, and follow-up. During this time, the last release of m-RESIST prototype was used and the content was frozen; therefore, no major changes were realized. Once patients received explanations about the study and signed the informed consent form (recruitment period), the study smartwatch and smartphone with the m-RESIST app preinstalled were given, and patients were trained to use the devices. Then, key elements of the m-RESIST intervention were performed (eg, treatment plan) (preintervention period). Next, the 3-month intervention was conducted, including 6 sessions with the mental health care providers, delivered every 15 days. Two visits were conducted face-to-face and 4 were held by videoconference. During this period, patients were recommended to use the smartwatch daily and to check the app at least once a day for questionnaires, recommendations, and reminders. At the end of the 3 months, the final visit was performed (follow-up period). The variables of feasibility, acceptability, usability, and satisfaction with the intervention were evaluated.

### Data Analysis

Statistical analyses were performed with the SPSS statistics (version 24; IBM Corp). Descriptive statistical analyses were performed to summarize the sociodemographic characteristics of the patients with TRS. Regarding the main variables, descriptive statistical analyses (means [SD]; frequencies and percentages) were performed to determine the feasibility (rates of willingness to participate, dropouts, and nonuse of the intervention), acceptability, usability for quantitative data, and satisfaction with the intervention. Differences in prescores and postscores of usability were analyzed using paired 2-sided *t* tests. Furthermore, in case of the usability variable, qualitative data were collected from the user experience questionnaire and the interval question. A qualitative thematic/content analysis was performed according to Mayring [26]. This method is a summary technique, where patterns in the text are found to look for themes in the data.

In terms of the operational criteria of willingness to enroll, nonusage, dropout, acceptability, usability, and satisfaction, there was no consensus to determine the cutoff points. For this reason, in this study, they were defined based on the criteria of the feasibility studies concerning the importance of establishing criteria aimed at the success of a randomized clinical trial [27,28]. Thus, high and demanding criteria were defined.

## Results

### Sample Population Characteristics

The final sample population in this pilot study consisted of 39 patients with TRS: 11 in Sant Pau Hospital, 14 in Semmelweis University, and 14 in Gertner Institute. No statistically significant differences in sociodemographic characteristics were found at baseline between the participants in the 3 sites. See Table 1 for a summary of the sociodemographic data. Regarding the degree of skill with ICT tools, around 75% (28/39) of the total sample of patients was distributed between the 2 intermediate levels (good and average levels).

**Table 1:** Baseline sociodemographic characteristics of the patients with treatment-resistant schizophrenia (N=39).

Characteristics		Values
Age (years), mean (SD)		33.6 (7.67)
**Gender, n (%)**		
	Women	17 (44)
	Men	22 (56)
**Ethnicity, n (%)**		
	Asian	9 (23)
	Caucasian	29 (74)
	Sub-Saharan Africa	1 (3)
**Marital status, n (%)**		
	Divorced/separated	2 (5)
	Married	4 (10)
	Single	30 (77)
	Stable relationship	2 (5)
	Widowed	1 (3)
**Cohabitation, n (%)**		
	Alone	4 (10)
	Sheltered accommodation	1 (3)
	With children	1 (3)
	With parents or relatives	27 (69)
	With flat mates (non–sheltered accommodation)	1 (3)
	With significant other	5 (13)
**Educational level, n (%)**		
	Primary school	18 (46)
	Secondary school	13 (33)
	University	8 (21)
**Occupation, n (%)**		
	Full-time job	10 (26)
	Part-time job	9 (23)
	Pensioner	6 (15)
	Sheltered job	2 (5)
	Student	1 (3)
	Unemployed	11 (28)

### Feasibility of the m-RESIST Intervention

**Figure 1:**
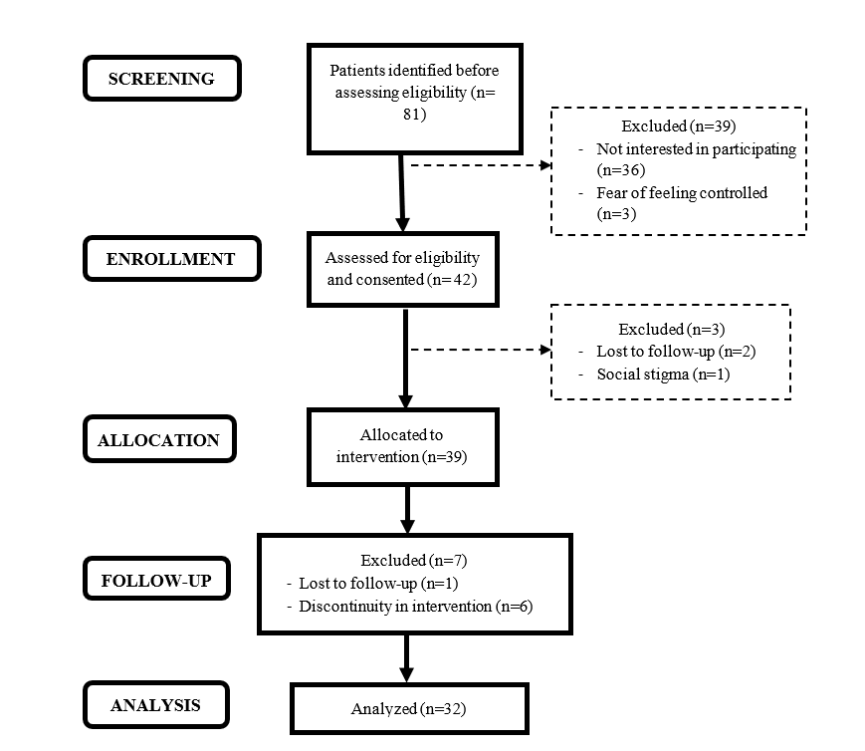
m-RESIST study participation flowchart based on the CONSORT (Consolidated Standards of Reporting Trials) 2010 statement recommendations.

The variables of willingness to participate, dropouts in the study, nonuse of the intervention, and patient compliance with the app were evaluated. The study participation flowchart based on the CONSORT (Consolidated Standards of Reporting Trials) 2010 statement recommendations [28] is presented in Figure 1. Approximately 52% (42/81) of the patients with TRS identified as candidates were receptive to participating in this study. With this result, the hypothesis was not confirmed (rate ≥70%). The reasons for not participating were lack of interest and fear of being controlled by electronic devices. Of the 39 patients who started the 3-month intervention period, 7 dropped out of the study, which represented a dropout rate of 18%. With this result, the hypothesis was not confirmed (rate <15%). Regarding the reasons for the withdrawal of patients, we grouped them into 4 main categories: (1) loss to follow-up, that is, not attending visits or answering calls; (2) clinical worsening, where hospitalization was required in 2 cases; (3) physical discomfort, mainly associated with the smartwatch, and (4) social stigma, that is, concern about showing technological devices in public that could be related to their diagnosis. All participants who completed the pilot study and participated in the follow-up (n=32) used the 2 m-RESIST tools (smartwatch and app) until the end of the study. The hypothesis of the study was fulfilled (nonuse rate <15%).

With regard to compliance of the patients, the rate of reading messages was 49.5% (895/1818) and the response rate was 13.6% (246/1818). Regarding the number of questionnaires that the patients received during the study and the degree of compliance with them, the response rate was close to 40%.

### Acceptability of the m-RESIST Intervention

The mean score for TAM dimensions on a scale from 1 (totally disagree) to 7 (totally agree) showed positive results in all dimensions (see Table 2).

Over 70% (22/32) of the patients considered that by using the m-RESIST intervention, health care professionals could provide better control of their conditions and they could receive more appropriate care. Over 80% (27/32) of the patients considered m-RESIST intervention as a user-friendly technology and easy-to-use; low perception of barriers was detected (12/32, 38%) and most of the patients (27/32, 84%) agreed with the existence of facilitators (81%-84%). Thus, the acceptability of the m-RESIST intervention was moderate (the tendency of response was near to a frequency of 75%), with 75% (24/32) of the patients showing a moderate-to-high intention to use the m-RESIST intervention. With this result, the hypothesis was not confirmed (>80%).

**Table 2:** Results of Technology Acceptance Model dimensions (n=32).

Dimension	Values, mean (SD)
Perceived utility	5.16 (1.14)
Perceived ease of use	5.39 (1.16)
Attitude	5.02 (1.10)
Barriers	3.84 (1.84)
Intention to use	5.19 (1.38)
Subjective norm^a^	5.34 (1.04)
Facilitators^b^	5.34 (1.08)

^a^Assesses the extent to which the potential adopter believes that people who are important to him or her will approve his or her adopting the system.

^b^Refers to the degree to which the potential adopter believes that an organizational and technical infrastructure exists to support the use of the system.

### Usability of the m-RESIST Intervention

#### Results of Closed Questions

The results of the usability analysis of the m-RESIST intervention were different between the app and the smartwatch. Over 80% (25/32) of the patients perceived the app as easy-to-use. It was considered easy to learn, clear, and understandable, and this perception increased over time (Multimedia Appendix 2). Over 65% of the patients found that the quality of the app’s content was good (eg, fonts, size, overall look, feel of the pages). Finally, over 65% of the patients considered the app to be well-integrated (24/32), recommended the app to others (19/32), and thought it would be good to use it in addition to the traditional treatment methods (22/32).

Regarding the smartwatch, a critical attitude prevailed about its ease of use and its usefulness in managing the disorder (Multimedia Appendix 2). Only over 40% (14/32) of the patients showed a positive attitude. At the end of the pilot study, over 60% (18/32) reported that they would recommend the device to others, but only 31% (10/32) reported that they would use it to manage their condition. As shown in Table 3, patients' experience with the digital tools was similar in both periods of time.

**Table 3:** User experience at periods Time 1 (middle pilot) and Time 2 (end of pilot) (n=32).^a^

		Time 1, mean (SD)	Time 2, mean (SD)	*t* *(df)*	*P* value
**App**					
	PEU^b^	16.81 (2.21)	16.45 (2.11)	1.10 (30)	.28
	PU^c^	12.32 (2.89)	11.39 (3.87)	1.30 (30)	.20
	PQC^d^	17.77(3.55)	17.45(3.87)	0.58 (30)	.57
	Attitude	21.26 (3.69)	20.97 (3.23)	0.40 (30)	.69
**Smartwatch**					
	PEU	17.23 (2.50)	17.39 (2.38)	–0.32 (30)	.75
	PU	13.39 (3.16)	13.58 (3.06)	–0.43 (30)	.67
	PQC	22.13 (5.43)	21.29 (5.77)	1.24 (30)	.23
	Attitude	21.94 (4.30)	22.65 (4.18)	–1.01 (30)	.32

^a^Scale scores from 1 (strongly agree; excellent) to 5 (strongly disagree; not know/no opinion).

^b^PEU: perceived ease of use.

^c^PU: perceived usefulness.

^d^PQC: perceived quality of content.

The usability of the m-RESIST intervention was medium for the app (the tendency of response was near to a frequency of 65%) and low for the smartwatch (the tendency of response was near to a frequency of 45%). With this result, the hypothesis was not confirmed (>80%).

#### Results of Open Questions

The first core theme was the feeling of security due to easier and quicker communication with professionals. The second theme was the perception of the m-RESIST intervention as promising and useful, and the step counter and the alarm button were evaluated better than other features. The third theme was the m-RESIST limitations. Patients mentioned technical difficulties with the system, mostly related to problems of synchronization and charge in the smartwatch and the mobile phone. Some patients also had other complaints about the smartwatch, such as the device irritated their skin and that there was lack of choice regarding the type of watch (color, size). Finally, patients in general communicated about being proud of being chosen as a participant for an ICT research project.

### Patient Satisfaction With the m-RESIST Intervention

Of the total sample, 78% (25/32) of the participants rated the quality of the service as excellent or good, 75% (24/32) felt they received the services they wanted, and 84% (28/32) reported that they would recommend the intervention program. Moreover, 94% (30/32) of the patients were very or mostly satisfied with the intervention and 84% (27/32) reported that they would use it again. Satisfaction with the m-RESIST intervention was generally good with a mean total score of 25 (SD 4.31) on the Client Satisfaction Questionnaire-8 item scale. With this result, the hypothesis was not confirmed (>80%).

### Safety

Regarding the safety of the study, the clinical status of the patient was monitored and evaluated based on the incidence of adverse events. Three serious adverse events were reported during this study that required hospitalization due to worsening of psychotic symptoms. No direct association between them and the m-RESIST intervention or the protocol procedures was found. The most probable causes were the evolution of the clinical pattern in 2 cases and a decrease in the sleep pattern in the other case.

## Discussion

### Principal Findings

Our novel findings indicate that the m-RESIST intervention is feasible and is an acceptable, satisfactory, and potentially useful tool for a population with TRS who have major clinical challenges with usually poor outcomes and low adherence to treatment.

### Feasibility of the m-RESIST Intervention

Most of the patients in our study had a high knowledge of ICT and owned a smartphone with an internet connection. This finding is similar to that reported in previous studies on populations with psychosis. According to a 2015 meta-analysis [9], smartphone ownership rate among patients with psychosis has increased proportionally in recent years (81%), and this rate is similar to that observed in the general population (90%). Moreover, the willingness rate to participate in this study was 52% (42/81), which was in line with that reported in other pilot studies on mobile app–based interventions for schizophrenia: 50% in the ClinTouch study [8], 40% in FOCUS [6], and 58% in PeerFIT [29]. Further, the dropout rate in our study was acceptable and similar to that reported in previous studies such as ClinTouch [8], PeerFIT [29], and CrossCheck [30]. However, some studies had almost no dropouts, for example, FOCUS [6], SleepSight [31], or App4Independence [15].

In future clinical trials, it is necessary to explore the actors and reasons for dropouts to diminish such instances and to adapt the digital intervention to the target populations. Of the pilot studies on psychosis included in 2 systematic reviews [12,32], only 3 examined the reasons for dropouts [6,29,33]. Laine et al [34] obtained high rates of refusal to participate (70%) and abandonment (48%), in contrast with the high compliance of the final sample. These findings suggest that digital technology can be used by patients with psychotic disorders. However, to incorporate digital health in patients’ treatments, apart from a receptive attitude, it is necessary to explore the degree of trust in technology. An interesting fact observed is that the initial reluctance toward using new technologies can be reversed: as the patient’s experience increases, a more receptive attitude is observed. Finally, the nonuse rate of the intervention in this study was observed to be low as in other mobile phone–based interventions, wherein all the patients who underwent the final evaluations in the studies used the devices until the end [8,33,35].

It was not possible to validly evaluate the compliance of the patients with this intervention. Errors in the app (eg, visualization of messages with errors in the characters, part of the message missing, errors in the notifications), mainly during the first middle part of the pilot study, resulted in patients’ inability to access the contents properly. Thus, results on compliance must be evaluated with caution.

### Acceptability, Usability, and Satisfaction of the m-RESIST Intervention

The m-RESIST digital intervention achieved a moderate level of acceptability, with 75% (24/32) of the patients showing moderate to high intention of using the intervention. The acceptability results are consistent with data shown in a meta-analysis [12], where the authors reported that around 60% of patients with psychotic disorders were in favor of using smartphones to monitor their mental health status. In general, most studies on patients with schizophrenia have shown their high acceptability toward digital interventions based on smartphones and aimed to (1) monitor variables (clinical symptoms, functioning, and mood) using the ecological momentary assessment methodology or other monitoring systems; (2) implement interventions to improve clinical symptoms and promote healthy lifestyle habits; and (3) facilitate contact with clinical professionals [12,34-36].

Regarding usability, the tendency of response toward the app was positive in 70% (22/32) of the patients. In previous mHealth pilot studies, similar values of usability were obtained [37,38]. It is important to note that most patients considered that the m-RESIST intervention should be used in addition to traditional treatments and not as a replacement. The patients feared losing face-to-face contact with the professional team and “being monitored by a machine.” The study of Baumel et al [39] would be a good example of how to add digitalization to the traditional treatment for schizophrenia. Baumel et al [39] developed a technology-based intervention for schizophrenia, which integrated digital tools and human support, and it achieved good outcomes in terms of retention (93% of the participants finished the program), acceptability, and satisfaction. Baumel et al [39] considered that the coach’s supportive role, providing technological guidance and delivering relapse prevention treatment, was one of the keys to patient adherence to their intervention.

In addition, it should be highlighted that previous mHealth studies aimed at people with schizophrenia used different methodologies to evaluate acceptability and usability. However, none of them have elaborated semistructured questionnaires to assess operational dimensions of acceptability, such as perceived ease of use or barriers. In this sense, it has not yet been possible to “capture the complex nature of acceptability” [40]. Berry et al [40] insist on the need to perform qualitative evaluations of acceptability during the different stages of a study. This same idea would also apply to the usability variable [41]. In the case of the m-RESIST study, semistructured interviews focusing on the evaluation of specific dimensions of acceptability and usability and based on current theoretical approaches (TAM, Living labs) were established.

Regarding satisfaction with the m-RESIST intervention, results showed that 78% (25/32) of the sample was quite to very satisfied with the service provided: 94% (30/32) of the sample was generally satisfied with the m-RESIST intervention and 84% (27/32) would use it again. This percentage is similar to that obtained in other digital interventions tailored for patients with schizophrenia (FOCUS [42], PEAR-004 [43], PeerFIT [44], WellWave [45]).

A noteworthy finding of our study is that patients distinguished the whole intervention from its components, that is, patients had a positive and satisfactory vision about the m-RESIST intervention (digital tools and tailored therapeutic program), but they had doubts about the functioning of the app and the smartwatch because they did not work properly during the pilots. Although a high percentage of patients reported that they would recommend the m-RESIST intervention (digital tools and tailored therapeutic program) and considered it to be a good idea to add the app and smartwatch to the treatment as usual, only 35% (11/32) reported that they would use these specific tools to manage their condition, while practically, the rest were undecided about the usability of the app and smartwatch to treat their own state of health. It is probably because patients appreciate the digital tools’ functionalities (eg, communication, steps counter, alarm button for emergencies) but could not test them properly due to technical problems. Thus, the m-RESIST intervention was valued as promising and it was rated successfully, but in terms of usability, it did not convince completely.

The added value of the m-RESIST intervention is to respond to the need of creating new care possibilities adapted to the specific nature of well-identified clinical subgroups such as patients with TRS [46]. The m-RESIST intervention has been designed to overcome fragmentation in providing care and to promote recovery from TRS. In the former case, the m-RESIST intervention favors continuity and immediacy of care by using digital tools to monitor patients’ state and detect individual early warning signs and ease communication with health care professionals. In the latter case, clinical, social, and personal recovery is promoted by the tailored therapeutic program delivered to patients with TRS (symptoms management-emotion regulation and healthy habits modules; see Multimedia Appendix 1).

### Strengths of This Study

This study has important strengths. First, this is the first time that digital technology has been incorporated for TRS intervention. This is important because apart from drugs, there are no specific programs of therapy available for this severe profile of patients. Second, we designed an intervention acceptable and with potential usability for TRS, with capacity for innovation at 2 levels: (1) methodological, created from a model where the patients with TRS and their needs are at the center [47] and (2) technological, intervention based on sensors, app, and web-based platform that would allow personalized follow-up and monitoring of patients based on their risk levels. Third, our study indicates that m-RESIST could be a safe digital intervention for patients with severe schizophrenia. Finally, we also developed an operational evaluation of acceptability and usability, performing interviews throughout different stages of the study.

### Limitations of This Study

There are some limitations in this study. First, the sample size was relatively small, although the size is similar to that reported in previous feasibility studies. Second, the presence of errors in the system operation, especially in communication and in questionnaires, made it impossible to assess the compliance of the participants. Third, the patients were not involved in the process of choosing the smartwatch, and the functioning of the smartwatch was also a limitation. An improvement in the whole process would have positively impacted the patient adherence and usability of this device.

### Conclusions

To date, no major progression is in sight for the treatment of schizophrenia and TRS [48], but small steps can be achieved, as shown in our study. The m-RESIST pilot study suggests that this intervention is a feasible treatment option for patients with TRS: easy to use, advisable, and with impact on the motivation for using it. The m-RESIST intervention has contributed to TRS treatment, thereby providing the basis of a new therapeutic program, which is based on novel technology, uses wearable computing solutions, and offers high modular and flexible functioning. For further development of integrated care tools, it will be crucial to involve health care providers and organizations. Thus, intervention programs based on ICTs will eventually be incorporated as an addition to the community services already existing in the mental health care routine.

## Appendix

Multimedia Appendix 1 m-RESIST tailored therapeutic program.

Multimedia Appendix 2 User experience questionnaire: open questions for the app and the smartwatch.

## Abbreviations

CONSORT: Consolidated Standards of Reporting Trials
ICT: information and communication technology
mHealth: mobile health
TAM: Technology Acceptance Model
TRS: treatment-resistant schizophrenia

## Data Availability

The data sets generated and analyzed during this study are available from the corresponding author on reasonable request.
