# Supplementary material for: m-RESIST, a Mobile Therapeutic Intervention for Treatment-Resistant Schizophrenia: Feasibility, Acceptability, and Usability Study
Source: JMIR Form Res. 2023 Jun 30;7:e46179. doi: 10.2196/46179 (PMC10365616; doi:10.2196/46179)
Supplement: Multimedia Appendix 1 [file formative_v7i1e46179_app1.docx]

# Multimedia Appendix

## m-RESIST tailored therapeutic program

The m-RESIST tailored therapeutic program was a modular and structured program with an assessment unit, a treatment unit, and a prediction of relapse unit.

### Section 1. The assessment unit

The assessment unit consisted of 3 questionnaires delivered by the app: Need 4-Help Scale (N4H), Risk Scale and Experience Scale. When the patient started the 3-months intervention it was considered that the Risk Level was 0 or Baseline (see Table S1). Subsequently, and depending on whether the system detected significant variations in its basal state, the N4H scale was sent (see Table S2).

Table S1- Definition of the Level of risk

| Punctuation | Level of risk | Examples of situations | Units of treatment |
| --- | --- | --- | --- |
| 0 | Baseline | No known risk. Normal functioning. | Basal Units^a^ |
| 1 | Low Risk | When a deviation from the baseline pattern is detected, but the patients say that they can cope with the situation by themselves | Basal Units |
| 2 | Medium Risk | The patients say that they need help from their mobile or informal caregiver | Risk Unit^b^ |
| 3 | High Risk | Patients say they need help from a healthcare professional | Risk Unit |
| 4 | Emergency | Life threat or security risk | Risk Unit |

^a^Basal Units: integrates 3 types of interventions, i.e. Symptom Management-Cognitive Behaviour Therapy (CBT), Adherence and Healthy Lifestyle units; ^b^Risk Unit: integrates one intervention, Symptom Management-Risk unit

N4H scale aimed to assess the need for intervention according to the patient's criteria to enhance empowerment and self-management. Moreover, it was answered from the patient's app, and each possible response to the questionnaire was associated with a predetermined level of risk. If, for example, the patient chose "I need help from my caregiver", the risk level raised to 2 (see Table S1).

Table S2- Need 4Help Scale

| Can cope | Need help from mobile | Need help from my caregiver | Need help from my doctor | Need urgent / emergency help |
| --- | --- | --- | --- | --- |

The Risk Scale identified and addressed risk situations (e.g. presence of auditory hallucinations) in a quickly and directly manner, providing information about them to health care professionals and sending personalized recommendations to patients and informal caregivers.

The Experience Scale was an ecological momentary assessment questionnaire addressed to monitor distress in real time and daily life, to help to identify the interaction between emotional experiences (i.e. how I feel right now) and contextual factors (e.g.- where I am, what I am doing), and to provide tailored recommendations to the specific patients’ situation.

### Section 2. The treatment unit

The treatment unit was based on the tailored identification of the main problems presented in patients with TRS. Four main problems were defined (see Table S2): risk behaviours (aggressive and suicide behaviour, and drug abuse), clinical symptoms (hallucinations, delusions and emotion dysregulation), adherence to pharmacological treatment and healthy lifestyle.

Table S3- List of problems

| Problem | Yes/No | Rate from 1 to 10 |
| --- | --- | --- |
| 1. Aggressive Behaviour  2. Suicidal Behaviour  3. Drug Abuse  4. Coping with hallucinations  5. Coping with delusions  6. Emotion dysregulation |  |  |
| 7. Adherence |  |  |
| 8. Healthy Lifestyle |  |  |

Based on this list of problems, a modular definition system was generated in order to select what type of intervention should be activated in each patient: symptom management-risk (problems 1-3), symptoms management-CBT (cognitive-behavioural therapy) (problems 4-6), treatment adherence (problem 7) or healthy lifestyle (problem 8). In case of detection of a risk situation by the Risk Scale, the symptoms management-risk intervention was activated automatically and the others interventions were deactivated.

### Section 3. Prediction of relapse unit

At the beginning of the study, and before starting the 3-months period, patients had to wear the smartwatch for a minimum period of 15 consecutive days. In this way, sufficient data were collected from the sensors to establish the baseline profile. Clinically significant deviation was defined as a 30% change below or above the reference value (baseline profile) over a 3-day period in one or more of the sensor parameters listed in Table S4. The Clinical Decision Support System was the m-RESIST functionality in charge to send notifications, questionnaires (e.g. the Need 4Help or the Risk Scale) or recommendations to patients when deviations were detected.

Table S4- List of parameters

| **Data** | **Function** |
| --- | --- |
| *GPS | Behavioural evaluation |
| Steps counter | Behavioural evaluation |
| Sleep pattern | Physiological evaluation |
| Heart rate | Physiological evaluation |

* *by setting this parameter, the m-RESIST system will just measure the time spent outside or at home.*
