# Supplementary material for: m-RESIST, a Mobile Therapeutic Intervention for Treatment-Resistant Schizophrenia: Feasibility, Acceptability, and Usability Study
Source: JMIR Form Res. 2023 Jun 30;7:e46179. doi: 10.2196/46179 (PMC10365616; doi:10.2196/46179)
Supplement: Multimedia Appendix 2 [file formative_v7i1e46179_app2.docx]

# Multimedia Appendix

User experience questionnaire: close questions for the app and the smartwatch

### App

| Items Usability | Time1 score, n (%) | | | | | Time2 score, n (%) | | | | |
| --- | --- | --- | --- | --- | --- | --- | --- | --- | --- | --- |
| **PEU**  **How easy is it to use the m-RESIST App? Agree or disagree.** | Strongly Agree | Somewhat Agree | NA/ND^a^ | Somewhat Disagree | Strongly Disagree | Strongly Agree | Somewhat Agree | NA/ND | Somewhat Disagree | Strongly Disagree |
| Learning how the m-RESIST App works is easy for me | 14 (44) | 8 (25) | 7 (22) | 2 (6) | 1 (3) | 16 (50) | 9 (28) | 5 (16) | 1 (3) | 0 (0) |
| I find it easy to get the m-RESIST App to do what I want to do | 10 (31) | 10 (31) | 7 (22) | 4 (13) | 1 (3) | 8 (25) | 13 (41) | 9 (28) | 1 (3) | 0 (0) |
| I find the m-RESIST App very difficult to use without a manual | 0 (0) | 3 (9) | 5 (16) | 13 (41) | 11 (34) | 1 (3) | 2 (6) | 2 (6) | 12 (38) | 14 (44) |
| I find that the m-RESIST App is designed for all levels of users | 9 (28) | 12 (38) | 4 (13) | 5 (16) | 2 (6) | 5 (16) | 15 (47) | 9 (28) | 2 (6) | 0 (0) |
| Interacting with the m-RESIST App is clear and understandable | 12 (38) | 14 (44) | 5 (16) | 1 (3) | 0 (0) | 10 (31) | 17 (53) | 3 (9) | 0 (0) | 1 (3) |
| I find the m-RESIST App unnecessarily complex | 0 (0) | 1 (3) | 5 (16) | 10 (3) | 16 (50) | 0 (0) | 0 (0) | 3 (9) | 14 (44) | 14 (44) |
| **PU**  **How useful do you find the m-RESIST App? Agree or disagree.** | Strongly Agree | Somewhat Agree | NA/ND | Somewhat Disagree | Strongly Disagree | Strongly Agree | Somewhat Agree | NA/ND | Somewhat Disagree | Strongly Disagree |
| Using the m-RESIST App will help me better manage my condition | 4 (13) | 13 (41) | 12 (38) | 3 (9) | 0 (0) | 4 (13) | 11 (34) | 11 (34) | 3 (9) | 2 (6) |
| The overview of appointments in a calendar is useful | 8 (25) | 9 (28) | 14 (44) | 0 (0) | 1 (3) | 10 (31) | 11 (34) | 9 (28) | 1 (3) | 0 (0) |
| The emergency button is useful | 9 (28) | 10 (31) | 11 (34) | 1 (3) | 1 (3) | 10 (31) | 13 (41) | 7 (22) | 1 (3) | 0 (0) |
| Receiving self-initiated questionnaires is useful | 4 (13) | 12 (38) | 10 (31) | 5 (16) | 1 (3) | 6 (19) | 9 (28) | 11 (34) | 4 (13) | 1 (3) |
| Access to psycho-educational material is helpful | 3 (9) | 2 (6) | 27 (84) | 0 (0) | 0 (0) | 8 (25) | 11 (34) | 9 (28) | 2 (6) | 1 (3) |
| **CONTENT**  **How would you rate the content of the m-RESIST App?** | Excellent | Good | Average | Poor | NK/NO^b^ | Excellent | Good | Average | Poor | NK/NO |
| Font and size (readability) of the words on the page | 11 (34) | 15 (47) | 6 (19) | 0 (0) | 0 (0) | 9 (28) | 14 (44) | 8 (25) | 0 (0) | 0 (0) |
| Appointment overview in the calendar | 5 (16) | 14 (44) | 3 (9) | 3 (9) | 7 (22) | 5 (16) | 17 (53) | 4 (13) | 1 (3) | 4 (13) |
| Overall look and feel of the pages | 6 (19) | 19 (59) | 7 (22) | 0 (0) | 0 (0) | 6 (19) | 15 (47) | 10 (31) | 0 (0) | 0 (0) |
| Terms used to describe the functions (e.g. messages, calendar) | 5 (16) | 18 (56) | 9 (28) | 0 (0) | 0 (0) | 5 (16) | 15 (47) | 11 (34) | 0 (0) | 0 (0) |
| Information richness | 2 (6) | 14 (44) | 11 (34) | 3 (9) | 2 (6) | 4 (13) | 14 (44) | 11 (34) | 1 (3) | 1 (3) |
| Position of items on the page | 6 (19) | 17 (53) | 8 (25) | 0 (0) | 1 (3) | 6 (19) | 12 (38) | 13 (41) | 0 (0) | 0 (0) |
| User Manual | 0 (0) | 3 (9) | 7 (22) | 2 (6) | 20 (63) | 0 (0) | 2 (6) | 7 (22) | 0 (0) | 15 (47) |
| **ATTITUDE**  **What is your attitude towards the m-RESIST App?** | Strongly Agree | Somewhat Agree | NA/ND | Somewhat Disagree | Strongly Disagree | Strongly Agree | Somewhat Agree | NA/ND | Somewhat Disagree | Strongly Disagree |
| I find the various functions in the App well integrated | 8 (25) | 17 (53) | 7 (22) | 0 (0) | 0 (0) | 7 (22) | 17 (53) | 6 (19) | 1 (3) | 0 (0) |
| I would recommend the App to others | 11 (34) | 12 (38) | 6 (19) | 3 (9) | 0 (0) | 10 (31) | 9 (28) | 10 (31) | 1 (3) | 1 (3) |
| Accessing the m-RESIST system through the App is convenient | 12 (38) | 13 (41) | 7 (22) | 0 (0) | 0 (0) | 12 (38) | 17 (53) | 2 (6) | 0 (0) | 0 (0) |
| I think that I would like to use the App to manage my condition | 5 (16) | 12 (38) | 10 (31) | 4 (13) | 1 (3) | 3 (9) | 8 (25) | 17 (53) | 2 (6) | 1 (3) |
| Unless I am obliged to use the App in some way, I see no reason to using it | 1 (3) | 4 (13) | 8 (25) | 11 (34) | 8 (25) | 1 (3) | 7 (22) | 8 (25) | 6 (19) | 9 (28) |
| I would pay a small fee for the App | 2 (6) | 8 (25) | 8 (25) | 10 (31) | 4 (13) | 4 (13) | 8 (25) | 8 (25) | 7 (22) | 4 (13) |
| I think it would be very good to use the App in addition to traditional methods | 7 (22) | 15 (47) | 5 (16) | 4 (13) | 1 (3) | 7 (22) | 15 (47) | 7 (22) | 2 (6) | 0 (0) |
| I think the App can replace traditional methods | 1 (3) | 2 (6) | 8 (25) | 10 (31) | 11 (34) | 1 (3) | 1 (3) | 10 (31) | 9 (28) | 10 (31) |

^a^NA/ND: Neither agree nor disagree; ^b^NK/NO: Not Know/No Opinion; Scale scores from 1 (Strongly agree/Excellent) to 5 (Strongly disagree/ Not Know/No Opinion)

### Smartwatch

| Items Usability | Time1 score, n (%) | | | | | Time2 score, n (%) | | | | |
| --- | --- | --- | --- | --- | --- | --- | --- | --- | --- | --- |
| **PEU**  **How easy is it to use the m-RESIST SW**^a^**? Agree or disagree.** | Strongly Agree | Somewhat Agree | NA/ND^b^ | Somewhat Disagree | Strongly Disagree | Strongly Agree | Somewhat Agree | NA/ND | Somewhat Disagree | Strongly Disagree |
| Learning how the m-RESIST SW works is easy for me | 9 (28) | 10 (31) | 7 (22) | 2 (6) | 4 (13) | 4 (12) | 15 (47) | 5 (16) | 4 (13) | 3 (9) |
| I find it easy to get the m-RESIST SW to do what I want to do | 6 (19) | 10 (31) | 12 (38) | 3 (9) | 1 (3) | 3 (9) | 10 (31) | 13 (41) | 4 (13) | 1 (3) |
| I find the m-RESIST SW very difficult to use without a manual | 2 (6) | 6 (19) | 10 (31) | 9 (28) | 5 (16) | 2 (6) | 8 (25) | 9 (28) | 8 (25) | 4 (13) |
| I find that the m-RESIST SW is designed for all levels of users | 6 (19) | 11(34) | 5 (16) | 8 (25) | 2 (6) | 3 (9) | 12 (38) | 7 (22) | 8 (25) | 1 (3) |
| Interacting with the m-RESIST SW is clear and understandable | 6 (19) | 12 (38) | 8 (25) | 2 (6) | 4 (13) | 3 (9) | 12 (38) | 8 (25) | 7 (22) | 1 (3) |
| I find the m-RESIST SW unnecessarily complex | 0 (0) | 3 (9) | 8 (25) | 11 (34) | 10 (31) | 0 (0) | 4 (13) | 10 (31) | 13 (41) | 4 (13) |
| **PU**  **How useful do you find the m-RESIST SW? Agree or disagree.** | Strongly Agree | Somewhat Agree | NA/ND | Somewhat Disagree | Strongly Disagree | Strongly Agree | Somewhat Agree | NA/ND | Somewhat Disagree | Strongly Disagree |
| Using the m-RESIST SW will help me better manage my condition | 6 (19) | 10 (31) | 7 (22) | 5 (16) | 4 (13) | 4 (12) | 10 (31) | 8 (25) | 7 (22) | 2 (6) |
| The overview of appointments in a calendar is useful | 5 (16) | 4 (13) | 21 (66) | 1 (3) | 1 (3) | 6 (19) | 4 (13) | 20 (63) | 1 (3) | 0 (0) |
| The emergency button is useful | 7 (22) | 3 (9) | 22 (69) | 0 (0) | 0 (0) | 4 (13) | 2 (6) | 25 (78) | 0 (0) | 0 (0) |
| Receiving self-initiated questionnaires is useful | 4 (13) | 7 (22) | 18 (56) | 2 (6) | 1 (3) | 2 (6) | 5 (16) | 22 (69) | 1 (3) | 1 (3) |
| Access to psycho-educational material is helpful | 2 (6) | 2 (6) | 28 (88) | 0 (0) | 0 (0) | 3 (9) | 3 (9) | 23 (72) | 1 (3) | 1 (3) |
| **CONTENT**  **How would you rate the content of the m-RESIST SW?** | Excellent | Good | Average | Poor | NK/NO^c^ | Excellent | Good | Average | Poor | NK/NO |
| Font and size (readability) of the words on the page | 4 (13) | 16 (50) | 6 (19) | 3 (9) | 3 (9) | 4 (13) | 16 (50) | 7 (22) | 3 (9) | 1 (3) |
| Appointment overview in the calendar | 0 (0) | 5 (16) | 5 (16) | 2 (6) | 20 (63) | 2 (6) | 7 (22) | 5 (16) | 2 (6) | 15 (47) |
| Overall look and feel of the pages | 9 (28) | 12 (38) | 7 (22) | 3 (9) | 1 (3) | 6 (19) | 11(34) | 8 (25) | 5 (16) | 1 (3) |
| Terms used to describe the functions (e.g. messages, calendar) | 1 (3) | 11(34) | 10 (31) | 3 (9) | 7 (22) | 1 (3) | 10 (31) | 9 (28) | 4 (13) | 7 (22) |
| Information richness | 4 (13) | 8 (25) | 11(34) | 2 (6) | 7 (22) | 4 (13) | 9 (28) | 9 (28) | 2 (6) | 7 (22) |
| Position of items on the page | 4 (13) | 10 (31) | 10 (31) | 1 (3) | 7 (22) | 5 (16) | 10 (31) | 11(34) | 2 (6) | 3 (9) |
| User Manual | 0 (0) | 5 (16) | 8 (25) | 1 (3) | 18 (56) | 0 (0) | 4 (13) | 8 (25) | 4 (13) | 15 (47) |
| **ATTITUDE**  **What is your attitude towards the m-RESIST SW?** | Strongly Agree | Somewhat Agree | NA/ND | Somewhat Disagree | Strongly Disagree | Strongly Agree | Somewhat Agree | NA/ND | Somewhat Disagree | Strongly Disagree |
| I find the various functions in the SW well integrated | 11(34) | 14 (44) | 7 (22) | 0 (0) | 0 (0) | 6 (19) | 14 (44) | 8 (25) | 3 (9) | 0 (0) |
| I would recommend the SW to others | 8 (25) | 12 (38) | 8 (25) | 3 (9) | 1 (3) | 7 (22) | 11(34) | 10 (31) | 3 (9) | 0 (0) |
| Accessing the m-RESIST system through the SW is convenient | 7 (22) | 15 (47) | 7 (22) | 3 (9) | 0 (0) | 4 (13) | 12 (38) | 10 (31) | 4 (13) | 1 (3) |
| I think that I would like to use the SW to manage my condition | 5 (16) | 13 (41) | 7 (22) | 3 (9) | 4 (13) | 1 (3) | 9 (28) | 15 (47) | 5 (16) | 1 (3) |
| Unless I am obliged to use the SW in some way, I see no reason to using it | 1 (3) | 7 (22) | 7 (22) | 10 (31) | 7 (22) | 1 (3) | 10 (31) | 5 (16) | 9 (28) | 6 (19) |
| I would pay a small fee for the SW | 3 (9) | 5 (16) | 8 (25) | 10 (31) | 6 (19) | 3 (9) | 7 (22) | 7 (22) | 8 (25) | 6 (19) |
| I think it would be very good to use the SW in addition to traditional methods | 7 (22) | 15 (47) | 7 (22) | 2 (6) | 1 (3) | 6 (19) | 13 (41) | 9 (28) | 3 (9) | 0 (0) |
| I think the SW can replace traditional methods | 0 (0) | 3 (9) | 6 (19) | 11(34) | 12 (38) | 1 (3) | 1 (3) | 10 (31) | 8 (25) | 11(34) |

^a^SW: Smartwatch; ^b^NA/ND: Neither agree nor disagree; ^c^NK/NO: Not Know/No Opinion; Scale scores from 1 (Strongly agree/Excellent) to 5 (Strongly disagree/ Not Know/No Opinion)
